# Optic nerve sheath diameter and optic nerve sheath diameter/eyeball transverse diameter ratio in prediction of malignant progression in ischemic stroke

**DOI:** 10.3389/fneur.2022.998389

**Published:** 2022-09-08

**Authors:** Yuan Guo, Yinjuan Chen, Chaoxiong Shen, Daofeng Fan, Xiaohong Hu, Jiaojiao Duan, Yangui Chen

**Affiliations:** Neurology Department, Longyan First Affiliated Hospital of Fujian Medical University, Longyan, China

**Keywords:** optic nerve sheath diameter, eyeball transverse diameter, intracranial pressure, secondary malignant middle cerebral artery infarction, ischemic stroke

## Abstract

**Background:**

The optic nerve sheath diameter (ONSD)/eyeball transverse diameter (ETD) ratio has been suggested in the evaluation of intracranial pressure (ICP). The aim of this study was to evaluate the predictive value of ONSD and ONSD/ETD in relation to risk for secondary malignant middle cerebral artery infarction (MMI).

**Methods:**

A total of 91 patients with MCA occlusion were included in this study. Data were divided into two groups based on development of MMI or not. ONSD and ETD were measured by unenhanced computed tomography (CT). The differences in ONSD and the ONSD/ETD ratios between the MMI and non-MMI groups were compared. Receiver operating characteristic curve analyses were used to test the diagnostic value of ONSD and ONSD/ETD independently, to predict MMI.

**Results:**

The ONSD in the MMI group and non-MMI group were 5.744 ± 0.140 mm and 5.443 ± 0.315 mm, respectively (*P* = 0.001). In addition, the ONSD/ETD ratios in the MMI group and non-MMI group were 0.258 ± 0.008 and 0.245 ± 0.006, respectively (*P* = 0.001). The receiver operating characteristic (ROC) curve demonstrated an area under the curve (AUC) for ONSD of 0.812 [95% confidence interval (CI): 0.718–0.906, *P* = 0.001], with a sensitivity of 97.4% and a specificity of 66.0% at the cut-off value of 5.520 mm. The AUC for ONSD/ETD ratio in predicting occurrence of MMI was 0.895 (95% CI: 0.823–0.968, *P* = 0.001), with a sensitivity of 84.2% and a specificity of 92.5% at a cut-off value of 0.250.

**Conclusion:**

In acute stroke patients with massive cerebral infarction, an increased ONSD or ONSD/ETD ratio increases the odds of malignant progression and may be used as an indicator for emergent therapeutic interventions. In addition, the ONSD/ETD ratio may be more valuable than ONSD in predicting the malignant progression of acute stroke patients.

## Introduction

Acute cerebral infarction is a common disease in neurology. The prognosis of patients is typically related to the infarct size and location. Partial or complete obstruction of the middle cerebral artery (MCA) leads to severe cerebral edema, increased intracranial pressure (ICP), midline displacement of brain tissue, and even the formation of cerebral hernia, otherwise known as malignant middle cerebral artery infarction (MMI). Usually, the most severe brain swelling develops within 1–5 days after stroke (1). Recent studies have shown that early rather than late decompressive interventions can improve clinical outcomes in patients at risk for secondary MMI (2, 3). The fatality rate can be as high as 70–80% if conservative medical treatment is adopted instead of active surgical intervention (4, 5). Therefore, the early identification of the patients who are likely to develop the MMI is crucial.

**Figure 1:**
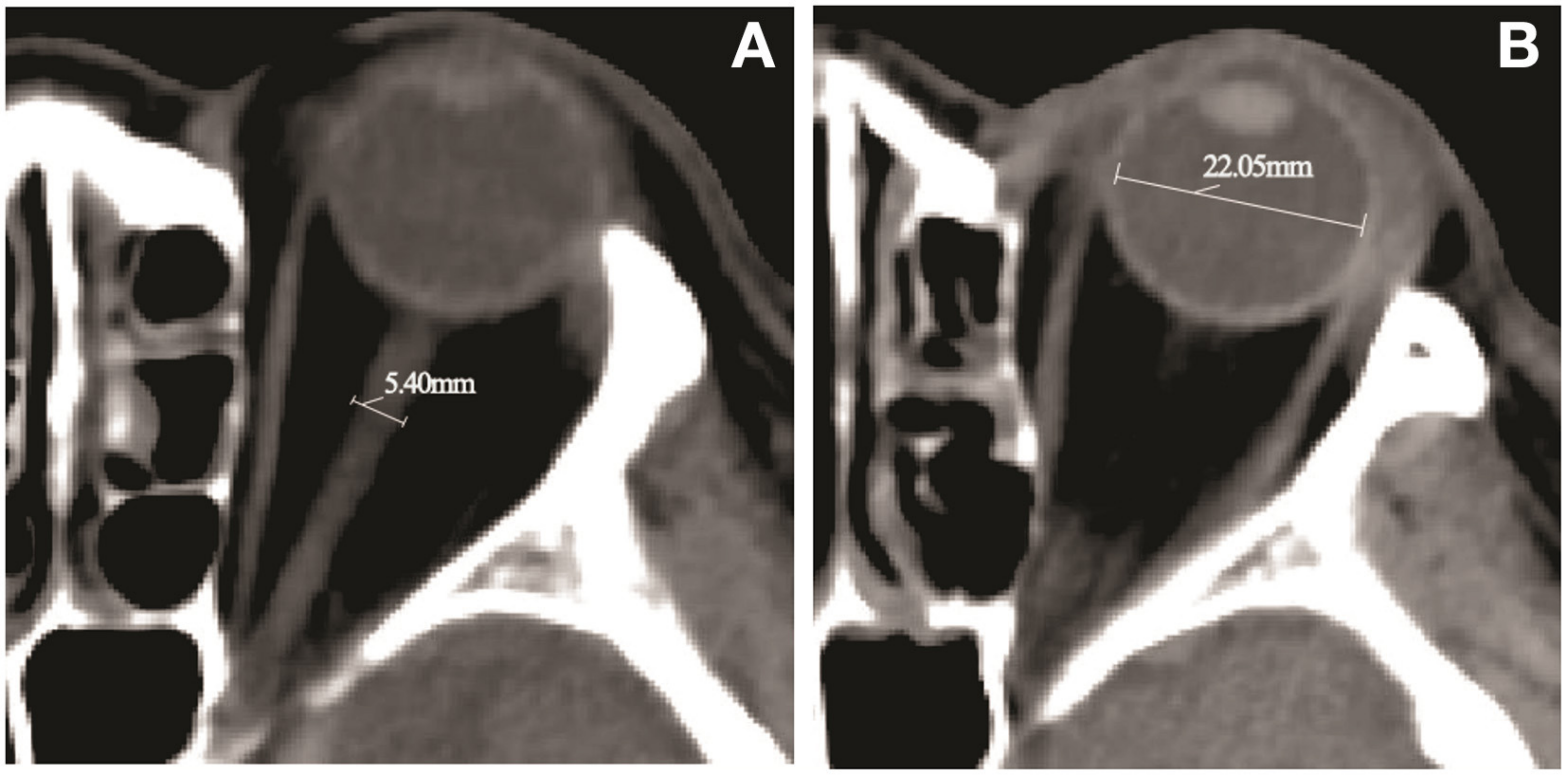
Computed tomography (CT) image of a 69-year-old female patient with a middle cerebral artery (MCA) infarction due to an occlusion of the proximal M1 segment. **(A)** The measurement of the optic nerve sheath diameter (ONSD) indicates diameter of 5.40 mm. **(B)** The eyeball transverse diameter (ETD) retina to retina measurement by head CT scan indicates diameter of 22.05 mm.

In patients with MMI, hemispheric brain swelling may lead to shifting brain tissue, while patients undergoing cerebral infarction may observe early elevation of ICP (6). The optic nerve, enveloped by cerebrospinal fluid and the arachnoid membrane, is an important component of the central nervous system. The subarachnoid space surrounding the optic nerve is connected with the intracranial mass, and the change in cerebrospinal fluid pressure can be transmitted along the optic nerve sheath. The presence of an increased optic nerve sheath diameter (ONSD) serves as an indirect marker of changes in ICP due to the direct influence of the ICP on the diameter of the subarachnoid space around the optic nerve (7). Studies assessing the ultrasound-based evaluation of the optic nerve have supported the notion that ONSD might accurately identify patients at risk for developing MMI (8, 9). However, ultrasound-based ONSD measurements require technical expertise to obtain adequate images, which limits its ubiquitous clinical utility. Alternatively, ONSD measurements obtained by computed tomography (CT) are strongly correlated with ICP in patients with brain injury and showed excellent agreement both between raters and between sides in the same patient (10, 11). The thickness of the optic nerve is proportional to the size of the eyeball (12). It has been suggested that the ratio of ONSD/eyeball transverse diameter (ETD) may thus reduce the variation of ONSD and provide an alternative measure for ICP monitoring, with more accurate results (13, 14). The measured parameters of ONSD and ETD can be referred to Figure 1. So far, few studies have evaluated the predictive value of ONSD and ONSD/ETD measured by CT for development of MMI.

The aim of this study was to evaluate the predictive value of ONSD and ONSD/ETD in relation to risk for secondary MMI in a cohort of patients with MCA infarction.

## Methods

### Patient selection

This study was approved by the ethics committee of Longyan First Affiliated Hospital of Fujian Medical University and performed according to the ethical standards of the Declaration of Helsinki. For the integrity of case data, we retrospectively collected all MCA infarction-related data available in our Neurology department from July 2016 to December 2021. Inclusion criteria were as follows: (1) ischemic stroke is listed as the primary diagnosis, identified using the International Classification of Diseases, 10th Revision, Clinical Modification (ICD-10CM) diagnostic codes I63, I64, I65, and I66; (2) age >18 years; (3) acute hemispheric infarction involving MCA region (covering more than two thirds of the MCA territory); (4) non-enhanced CT scan and computed tomography angiography (CTA) scan were performed in the emergency room; (5) availability of CT imaging 12–36 h after stroke onset; (6) MCA main trunk occlusion with or without internal carotid artery (ICA) occlusion confirmed on CTA images; (7) infarction confirmed by CT scan; (8) availability of follow-up CT imaging. Exclusion criteria were: (1) the patients with the diagnoses of posterior intracranial circulation occlusion (ICD-10CM diagnostic codes G45.0, I65.0, I65.1, I66.3, and I63.904); (2) age <18 years; (3) previous ocular pathology (as glaucoma or cataract) and optic nerve diseases (ICD-10CM diagnostic codes H26, H40, H46, H47); (4) concurrent hemorrhagic stroke (ICD-10CM diagnostic codes I60, I61); (5) concurrent vascular territory infarction other than MCA; (6) other comorbidities that affect the state of the nervous system, such as seizures or acute respiratory distress syndrome.

The study screened 286 patients by ICD code, and a total of 91 patients were included in the final analysis after further screening by inclusion criteria and exclusion criteria. Data were divided into 2 groups based on patients who had or had not developed MMI, hereto referred as the MMI group and the non-MMI group. Diagnosis of MMI was defined as in Thomalla's study (15) according to the following criteria: (1) secondary neurological deterioration including decline of consciousness by 1 or more points on the level of consciousness item of the National Institutes of Health Stroke Scale (NIHSS) and (2) large space-occupying MCA infarction on follow-up CT (covering more than two thirds of the MCA territory with compression of ventricles or midline shift) assessed in consensus by an experienced neurologist and neuroradiologist.

### Baseline data

The following data were collected: gender, age, smoking, and drinking history, history of diabetes mellitus, hypertension, heart disease, Hyperlipidemia, C-reactive protein (CRP), D-dimer, NIHSS score, and latency between CT scan and stroke onset.

### ONSD and ETD measurement

All CT images were obtained with a 64-slice CT scanner (Siemens, Munich, Germany), with a single slice section of 0.6 mm. To analyze CT-based factors for the prediction of impending herniation, CT was taken 12–36 h after onset of cerebral infarction. The ONSD and ETD were measured using the middle third spine window (window width 60, window level 360), with identical contrast and brightness. The ONSD was measured 10 mm behind the globe, perpendicular to the linear axis of the optic nerve (Figure 1). The ETD was defined as the maximal transverse diameter of the eyeball from retina to retina (Figure 1). The values were averaged from measurements independently obtained by two neuroradiologists. All of the measurements were performed bilaterally, and the mean value was used to calculate the ONSD/ETD ratio.

### Statistical analysis

The analyses of the data were performed using IBM SPSS Statistical 20.0 software (IBM Corporation, NY, USA). Normally distributed continuous variables were expressed as mean ± standard deviation (SD) and the differences were analyzed using Student's *t-*tests. Categorical variables were expressed as percentages and the differences were analyzed using chi-square tests. The optimal threshold values of the ONSD and ONSD/ETD ratio for predicting MMI were determined by the area under the curve (AUC) of receiver operating characteristic (ROC) curve, and their sensitivity and specificity were calculated. A *P*-value of 0.05 was considered statistically significant.

## Results

### Baseline characteristics

A total of 38 MMI patients and 53 non-MMI patients were included in this study. The demographic and clinical characteristics of the MMI patients and non-MMI patients are shown in Table 1. The gender composition, number of smokers, drinkers, rates of diabetes mellitus, hypertension, atrial fibrillation, hyperlipidemia, levels of C-reactive protein, D-dimer, admission NIHSS score and latency between CT scan and stroke onset were not significantly different between MMI patients and non-MMI patients (*P* >0.05). Patients developing MMI were younger (mean age 62.68 ± 6.68 vs. 66.75 ± 9.26 years, *P* = 0.023).

**Table 1:** Baseline characteristics.

**Characteristics**	**MMI group (*n* = 38)**	**Non-MMI group (*n* = 53)**	***P*-value**
Male, *N* (%)	24 (63.16)	26 (49.06)	0.182
Mean age, years	62.68 ± 6.68	66.75 ± 9.26	0.023
Smoke, *N* (%)	15 (39.47)	14 (26.42)	0.187
Alcohol consumption, *N* (%)	15 (39.47)	20 (37.74)	0.867
Diabetes mellitus, *N* (%)	16 (42.10)	23 (43.40)	0.902
Hypertension, *N* (%)	22 (57.89)	27 (50.94)	0.512
Atrial fibrillation, *N* (%)	5 (11.16)	12 (22.64)	0.252
Hyperlipidemia, *N* (%)	19 (50.00)	29 (54.72)	0.657
C-reactive protein, mg/L	3.84 ± 6.2	5.69 ± 8.52	0.258
D-dimer, mg/L	0.66 ± 0.97	0.61 ± 0.54	0.786
Admission NIHSS score	17.66 ± 2.40	17.58 ± 1.56	0.870
Latency between CT scan and stroke onset, h	22.13 ± 4.76	23.17 ± 5.84	0.370

MMI, malignant middle cerebral artery infarction; NIHSS, national institutes of health stroke scale; CT, computed tomography.

### Measurements of ONSD and ETD

The ONSD in the MMI group and non-MMI group were 5.744 ± 0.140 mm and 5.443 ± 0.315 mm, respectively (*P* = 0.001). In addition, the ONSD/ETD ratios in the MMI group and non-MMI group were 0.258 ± 0.008 and 0.245 ± 0.006, respectively (*P* = 0.001). The parameters are detailed in Table 2.

**Table 2:** ONSD and ETD measurements by CT and the ONSD/ETD ratio in patients.

**Variables**	**MMI group (*n* = 38)**	**Non-MMI group (*n* = 53)**	***P-*value**
ONSD (mm)	5.744 ± 0.140	5.443 ± 0.315	0.001
ETD(mm)	22.307 ± 0.819	22.168 ± 0.974	0.476
ONSD/ETD	0.258 ± 0.008	0.245 ± 0.006	0.001

MMI, malignant middle cerebral artery infarction; ONSD, optic nerve sheath diameter; ETD, eyeball transverse diameter; CT, computed tomography.

### Predictive efficiency of ONSD/ETD ratio for MMI

**Figure 2:**
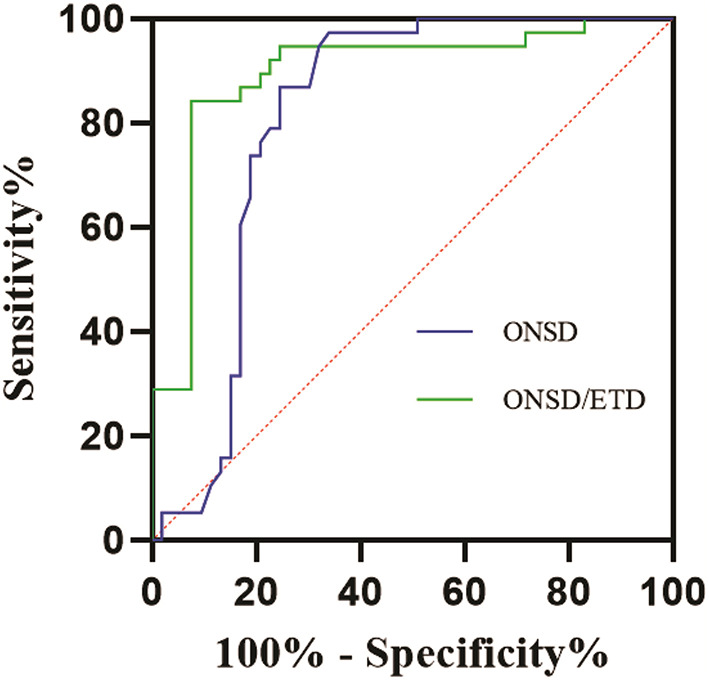
ROC curves for the efficiency of ONSD and ONSD/ETD ratio in predicting occurrence of MMI. ROC, receiver operating characteristic; MMI, malignant middle cerebral artery infarction; ONSD, optic nerve sheath diameter; ETD, eyeball transverse diameter.

The efficiency of ONSD and ONSD/ETD ratio in predicting the occurrence of MMI is shown in Figure 2. The ROC curve demonstrated an AUC for ONSD in predicting the occurrence of MMI was 0.812 [95% confidence interval (CI): 0.718–0.906, *P* = 0.001], with a sensitivity of 97.4% and specificity of 66.0% at a cut-off value of 5.520 mm. The AUC for ONSD/ETD ratio in predicting the occurrence of MMI was 0.895 [95% (CI): 0.823–0.968, *P* = 0.001], with a sensitivity of 84.2% and a specificity of 92.5% at a cut-off value of 0.250.

## Discussion

Patients with MMI have a poor prognosis due to space-occupying and life-threatening edema formation in the brain. Identifying patients at risk of developing fatal edema is critical for the earliest performance of decompressive hemicraniectomy. At present, there is a lack of clinical tools that can effectively predict the occurrence of malignant cerebral infarction. While many studies have attempted the early prediction of MMI, the gold standard for measuring ICP remains invasive monitoring. One study has even suggested that ICP monitoring is of little value in the vast majority of patients with acute ischemic stroke (16). On the other hand, infarct volume has been demonstrated as a reliable predictor of MMI (17). This study found that patients with MMI were more likely to be younger than those without MMI. One explanation may be rooted in the shrinkage of brain volume that increases with age, allowing greater space for brain swelling.

The ONSD is a reliable, non-invasive radiological marker of ICP, whether measured by magnetic resonance imaging (MRI), ultrasound or CT scan (11, 14, 18). Liu et al. (11) suggest that ONSD can reliably predict the requirement for surgery in patients with traumatic brain injury following admission to the emergency department (AUC = 0.920, 95% CI, 0.877–0.962). Similarly, Goel et al. (19) reported that ONSD can predict surgical intervention with a sensitivity and specificity of 98.3 and 62.5%, respectively. Even in non-traumatic cases, Amini et al. (20) reported that a cut-off value of 5.5 mm could be used to detect increased ICP with a sensitivity and specificity of 100%.

Due to the strong correlation between ONSD and ETD (12), the ONSD/ETD ratio has been introduced as a surrogate metric of ONSD accounting for interindividual variability due to orbit size (13, 21). Several researches have reported that changes in the ONSD/ETD ratio are more effective than ONSD in detecting increased ICP (13, 14, 22, 23). Albert et al. (24) found that an ONSD of more than 5.25 mm and an ONSD/ETD ratio of more than 0.232 on initial CT may identify MCA stroke patients at high risk of developing malignant MCA syndrome. Lee et al. (22) similarly described that the rate of ONSD/ETD changes can predict late malignant progression and midline shifting. The ONSD/ETD ratio may even reliably predict intracranial hypertension in traumatic brain injury patients (14).

Current research suggests that standard deviation of the ONSD measurements varies from 0.62 to 1.51, while the standard deviation of the ONSD/ETD index is 0.01–0.02, yielding more precise, normative data (13). Standard procedures involve measuring the ONSD from 3 mm behind the globe, though new studies suggest that for ICP monitoring, the most stable results can be obtained if the diameter is measured 10 mm from the globe (13). The rationale is that this depth is shielded from affects by tremor, gaze deviations, and involuntary movements of the eyes after trauma or stroke. To determine the value of the ONSD/ETD ratio in predicting, MMI, we performed a single-center retrospective cohort study.

In this study, ONSD was measured 10 mm behind the globe, and ROC curve was used to analyze the predictive value of ONSD/ETD for MMI. The results showed that the ONSD in the MMI group was 5.744 ± 0.140 mm, compared to 5.443 ± 0.315 mm in the non-MMI group. In addition, the ONSD/ETD ratio in the MMI group was 0.258 ± 0.008, compared to 0.245 ± 0.006 in the non-MMI group. The ROC curve demonstrated an AUC for ONSD in predicting the occurrence of MMI was 0.812 (95% CI: 0.718–0.906, *P* = 0.001), with sensitivity of 97.4% and specificity of 66.0% at a cut-off value of 5.520 mm. The AUC for the ONSD/ETD ratio in predicting the occurrence of MMI was 0.895 (95% CI: 0.823–0.968, *P* = 0.001), with sensitivity of 84.2% and specificity of 92.5% at a cut-off value of 0.250. The main finding of this study was that ONSD/ETD was an effective predictor of the development of MMI, with a higher accuracy than ONSD alone. The ONSD/ETD was also a much earlier predictor of ICP than the CT findings of cisternal effacement, sulcal effacement, ventricular compression, and cerebral herniation. Thus, ONSD/ETD measured on CT could serve as a non-invasive predictor of intracranial hypertension in patients, allowing for non-invasive monitoring that can be used in therapeutic decision making.

One of the limitations of this study was its single-center, retrospective design, which yielded a small sample size that was more vulnerable to bias. Second, invasive ICP monitoring was not performed as a control intervention. This is due to current practice guidelines, which do not recommend invasive ICP monitoring of ischemic stroke patients (25). Lastly, while we assessed the value of the ONSD/ETD ratio in predicting MMI, the relationship between ONSD/ETD and long-term ischemic stroke outcomes remain uncertain.

## Conclusion

In acute stroke patients with massive cerebral infarction, an increased ONSD or ONSD/ETD ratio may signal the increased odds of malignant progression, and may be used as an indicator for patients who may more likely benefit from emergent therapeutic interventions. We also report that the ONSD/ETD ratio may yield more clinical value than traditional ONSD in detecting elevated ICP and predicting the malignant progression of acute stroke patients. A multicenter study including different imaging devices with a larger sample size is necessary to confirm our results.

## Data availability statement

The raw data supporting the conclusions of this article will be made available by the authors, without undue reservation.

## Ethics statement

The studies involving human participants were reviewed and approved by the Longyan First Hospital Affiliated to Fujian Medical University. The patients/participants provided their written informed consent to participate in this study.

## Author contributions

YG, YiC, and CS designed and performed the experiments and wrote the manuscript. XH, JD, DF, and YaC collected and analyzed the data. All authors have read and approved the manuscript.

## Funding

This study was sponsored by Longyan City Science and Technology Plan Project (Grant No. 2020LYF17030). This funding supported the data collection, analysis and patient follow up.

## Conflict of interest

The authors declare that the research was conducted in the absence of any commercial or financial relationships that could be construed as a potential conflict of interest.

## Publisher's note

All claims expressed in this article are solely those of the authors and do not necessarily represent those of their affiliated organizations, or those of the publisher, the editors and the reviewers. Any product that may be evaluated in this article, or claim that may be made by its manufacturer, is not guaranteed or endorsed by the publisher.
